# Future Perspectives in Percutaneous Treatment of Tricuspid Regurgitation

**DOI:** 10.3389/fcvm.2020.581211

**Published:** 2020-10-14

**Authors:** Antonio Mangieri, Matteo Pagnesi, Damiano Regazzoli, Alessandra Laricchia, Edwin Ho, Ythan Goldberg, Mei Chau, Francesco Gallo, Andrea Fisicaro, Arif Khokhar, Antonio Colombo, Francesco Giannini, Azeem Latib

**Affiliations:** ^1^Cardiovascular Department, GVM Care and Research, Maria Cecilia Hospital, Cotignola, Italy; ^2^San Raffaele Scientific Institute, Milan, Italy; ^3^Unit of Cardiovascular Interventions, Humanitas Research Hospital, Rozzano, Italy; ^4^Department of Cardiology, Montefiore Medical Center, New York, NY, United States; ^5^Department of Cardiac Surgery, Montefiore Medical Center, New York, NY, United States

**Keywords:** transcatheter tricuspid valve intervention, transcatheter tricuspid valve replacement, tricuspid valve, tricuspid regurgitation, intracardiac echocardiography, transesophageal echocardiography

## Abstract

Tricuspid regurgitation (TR) has a not negligible prevalence and its severity is correlated with poorer outcomes. However, surgical options are rarely offered to these patients because of their high surgical risk. Given that medical therapy plays a limited role in the management of these patients, there is an increasing clinical need for transcatheter treatment options. Although, transcatheter tricuspid valve interventions (TTVIs) are still at an early stage, emerging data suggests their clinical effectiveness and safety, with preliminary results highlighting the potential benefits of transcatheter treatments over medical therapy. In this review, we highlight the challenges and future directions of current and emerging technologies dedicated to the treatment of TR along with an analysis of the next steps required in future clinical trials and studies dedicated to the treatment of the forgotten valve.

## Global Burden of Tricuspid Regurgitation and its Future Importance

Heart failure is an increasing public health issue with an overall prevalence of >37.7 million people worldwide (1). A wide range of cardiac conditions, hereditary defects, and systemic diseases can lead to heart failure, nevertheless valvular heart disease still represents one of the main causes (2). In 2010, ~106,000 valve surgeries were performed in the United States of America, however surgery was not possible in a significant proportion of patients due to comorbidities accounting for an elevated mortality risk (3). Consequently, minimally invasive percutaneous treatments for valvular diseases, which mitigate the risk of cardiac surgery, are gaining attraction. This concept is particularly true for tricuspid regurgitation (TR), the forgotten valvulopathy, which has been repeatedly shown to be associated with poor clinical outcomes, irrespective of the subgroups studied (4). In both men and women aged >70 years, the prevalence of moderate and severe TR reaches up to 1.5 and 5.6%, respectively while amongst patients with heart failure and reduced ejection fraction, the prevalence of moderate-severe TR is 26% and independently affects the prognosis (5, 6). Despite the increasing prevalence, isolated tricuspid valve (TV) repair or replacement is rarely performed nowadays due to poor results in terms of survival benefit and procedural mortality (7, 8). New transcatheter options, which offer a less invasive alternative to surgery, offer a potential alternative for these high-risk patients (4). Although no randomized controlled trials currently exist demonstrating the superiority of new TTVIs over medical therapy, emerging data suggest that new transcatheter therapies can improve patient's prognosis and quality of life, thus leading to a reduction in the use of diuretics and organ damage related to venous blood stasis (9).

## Clinical Trials in Tricuspid Intervention: What are the Next Steps?

Percutaneous treatment of TR represents a novel and rapidly growing field in interventional cardiology that poses unique challenges, which distinguish it from the treatment of left-sided valvulopathies. In the near future, percutaneous interventions for TR may offer an effective and low-risk solution to treat a large proportion of patients with symptomatic significant TR, ideally before the development of clinically advanced right heart failure (absence of extreme right ventricular dilation or dysfunction, advanced secondary renal or hepatic damage, cardiac cachexia, or severe refractory symptoms).

**Figure 1:**
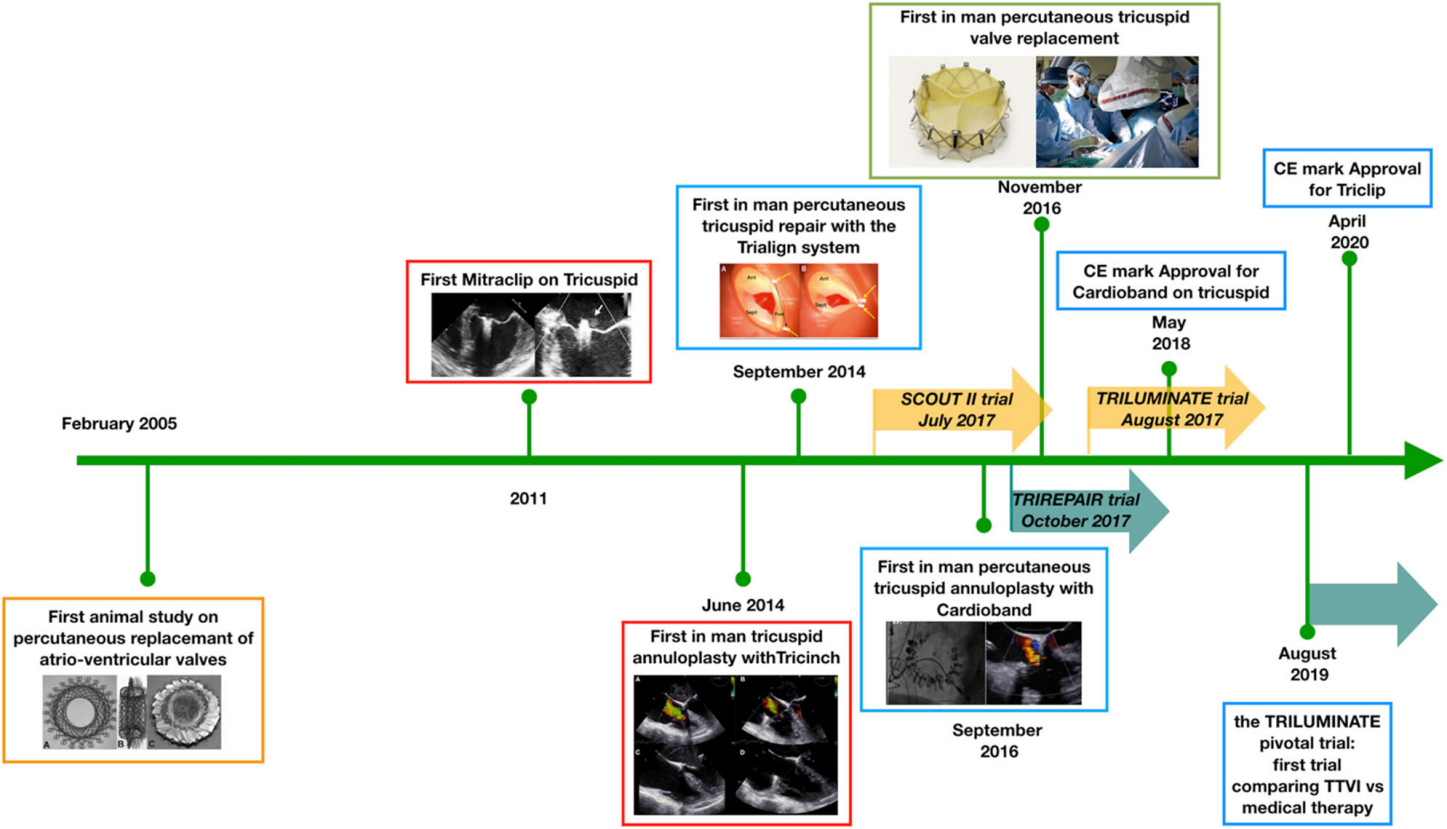
Developmental timeline of percutaneous tricuspid valve interventions. Since 2005, a number of technologies have been developed to address the problem of tricuspid regurgitation. At present, the first randomized controlled trials are enrolling patients to establish the potential effectiveness of percutaneous treatments over medical therapy. TTVI, transcatheter tricuspid valve intervention; CE, Conformité Européene.

In the last 15 years, significant progress has been made in developing the field of TTVIs (Figure 1) with numerous first-in-man studies and randomized controlled trials being initiated (Table 1). We have highlighted some of the important hurdles, which still need to be overcome in future studies focusing on TR interventions:

- **Comparing percutaneous tricuspid valve interventions to medical therapy**. Emerging data suggests that TTVI could be superior to medical therapy (9), but future studies are required to confirm this finding. The Clinical Trial to Evaluate Cardiovascular Outcomes In Patients Treated With the Tricuspid Valve Repair System (TRILUMINATE) Pivotal trial (NCT03904147) is currently enrolling 700 patients randomized 1:1 to medical therapy vs. TTVR using the TriClip (Abbott Vascular, Santa Clara, California). The trial is designed to demonstrate the safety and effectiveness of the TriClip device in improving clinical outcomes in symptomatic patients with severe TR. This trial will provide key insights into the potential effectiveness of percutaneous valve repair compared to medical therapy in terms of improving patient outcomes. Similarly, the Edwards PASCAL Transcatheter Valve Repair System Pivotal Clinical Trial (CLASP II TR trial, NCT 04097145) will randomize 825 patients with severe TR in a 2:1 fashion to receive either percutaneous treatment with the PASCAL device or medical therapy. The aim is to compare the effectiveness and potential superiority of the minimally invasive compared to a conservative approach in terms of mortality, heart failure hospitalization, need for subsequent surgery on the TV, and improvement in quality of life (Table 1). Of note, specific medical therapy could be useful in reducing TR severity or TR-derived symptoms and systemic venous congestion (e.g., aggressive diuretic therapy and/or rhythm control strategies for atrial fibrillation); this aspect is particularly relevant since future trials investigating TV interventions should apply standardized definitions of “optimal medical therapy” for TR, similarly to the concept of guideline-directed medical therapy applied in pivotal trials in the mitral regurgitation field.
- **Comparing percutaneous tricuspid valve interventions to surgery**. Patients affected by TR are usually considered at high surgical risk. Despite technical improvements, contemporary surgical mortality for isolated tricuspid regurgitation remains around 8.8% and has not declined over the years (7). Moreover, patients who undergo isolated TV replacement have a poor prognosis after surgery with a median survival of 1.2 years (10). Future trials comparing TTVI against surgery in patients not deemed at prohibitive surgical risk are warranted to establish if minimally-invasive treatments are a viable alternative to open-heart surgery in reducing mortality. Nevertheless, a trial comparing open-heart surgery vs. TTVI needs to be meticulously designed to assure an ethical treatment which does give any harm to the surgical group. For this reason, medical therapy could be a better comparator for this patients population which has a high mortality in case of open-heart surgery.
- **Need for standardization of clinical and procedural endpoints**. Across the field of aortic and mitral interventions, standardized clinical and procedural definitions have been established. In contrast, a detailed and specific consensus on endpoint definitions for TTVI is still not available and is urgently needed to clarify which procedural and clinical outcomes should be prioritized in this field (including a tailored assessment of symptoms and clinical status), and to ensure a standardized reporting of endpoints in future studies.
- **What is a “good result” in tricuspid intervention?** In patients treated with transcatheter aortic valve replacement (TAVR), data exists demonstrating that even residual mild paravalvular leak can have a detrimental effect on patient's survival (11). Similarly, following MitraClip therapy, residual moderate mitral regurgitation compared to ≤1+ mitral regurgitation was associated with worse follow-up outcomes including survival, symptom relief, and mitral regurgitation recurrence (12). In the tricuspid field, patients with residual significant TR still derive a symptomatic benefit from the intervention despite a perceived suboptimal result; however, preliminary data suggest that procedural failure (residual significant TR) is associated with worse prognosis at follow-up (higher risk of mortality and hospitalization for heart failure) (9, 13). Future studies specifically focusing on this topic are hugely needed.

**Table 1:** Current and future studies on percutaneous tricuspid valve interventions.

**Type of study**	**Name of the study**	**Device**	**Type of device**	**Clinical trial number**	**N of patients**	**Primary endpoints**
First in man	Early Feasibility Study of the Cardiovalve System for Tricuspid Regurgitation	Cardiovalve	Transcatheter valve	NCT04100720	15	Performance endpoint Successful access, delivery and retrieval of the delivery system
Post-market, prospective	Transcatheter Repair of Tricuspid Regurgitation With Edwards Cardioband TR System Post-Market Study (TriBAND)	Cardioband	Complete annuloplasty	NCT03779490	150	Procedure success Reduction in severity of Tricuspid Regurgitation at discharge
First in man	Study of Transcatheter Tricuspid Annular Repair (STTAR)	Minimally Invasive Annuloplasty Device	Complete annuloplasty	NCT03692598	60	Major adverse events Death, Q-wave MI, cardiac tamponade, cardiac surgery for failed device implantation, or stroke
First in man in USA	Edwards Cardioband Tricuspid Valve Reconstruction System Early Feasibility Study	Cardioband	Complete annuloplasty	NCT03382457	35	Freedom from device or procedure-related adverse events
First in man	TRICUS STUDY Euro - Safety and Efficacy of the TricValve® Device	TricValve	Heterotopic valve	NCT04141137	35	Percentage of participants with major adverse events KCCQ changes at 30 days
First in man	Edwards CLASP TR EFS	PASCAL	Leaflet plasty device	NCT03745313	65	Freedom from device or procedure-related adverse events
Randomized controlled trial	Edwards PASCAL Transcatheter Valve Repair System Pivotal Clinical Trial (CLASP II TR)	PASCAL vs. medical treatment	Leaflet plasty device	NCT04097145	825	Comparison of a composite endpoint (mortality, heart failure hospitalization, need for surgery on the tricuspid valve, and improvement of quality of life)
Randomized controlled trial	TRILUMINATE Pivotal Trial	TriClip vs. medical treatment	Leaflet plasty device	NCT03904147	700	Hierarchical composite of number of all-cause mortality or tricuspid valve surgery, heart failure hospitalizations, and quality of life assessment (KCCQ)
First in man study	Early Feasibility Study of the Percutaneous 4Tech TriCinch Coil Tricuspid Valve Repair System	TriCinch	Indirect annuloplasty system	NCT03632967	15	All-cause mortality of the Per Protocol cohort at 30 days post-procedure
First in man study	Early Feasibility Study of the Edwards EVOQUE Tricuspid Valve Replacement System	Evoque	Transcatheter tricuspid valve	NCT04221490	Unknown	Unknown
First in man study	FiH Study of the DaVingi™ TR System in the Treatment of Patients With Functional Tricuspid Regurgitation	DaVingi	Complete annuloplasty system	NCT03700918	15	Safety-the incidence and severity of device-related serious adverse device effects Device performance

*MI, myocardial infarction; KCCQ, Kansas city cardiomyopathy questionnaire*.

## Which Device for Which Patient? Moving Toward a Tailored Approach

**Figure 2:**
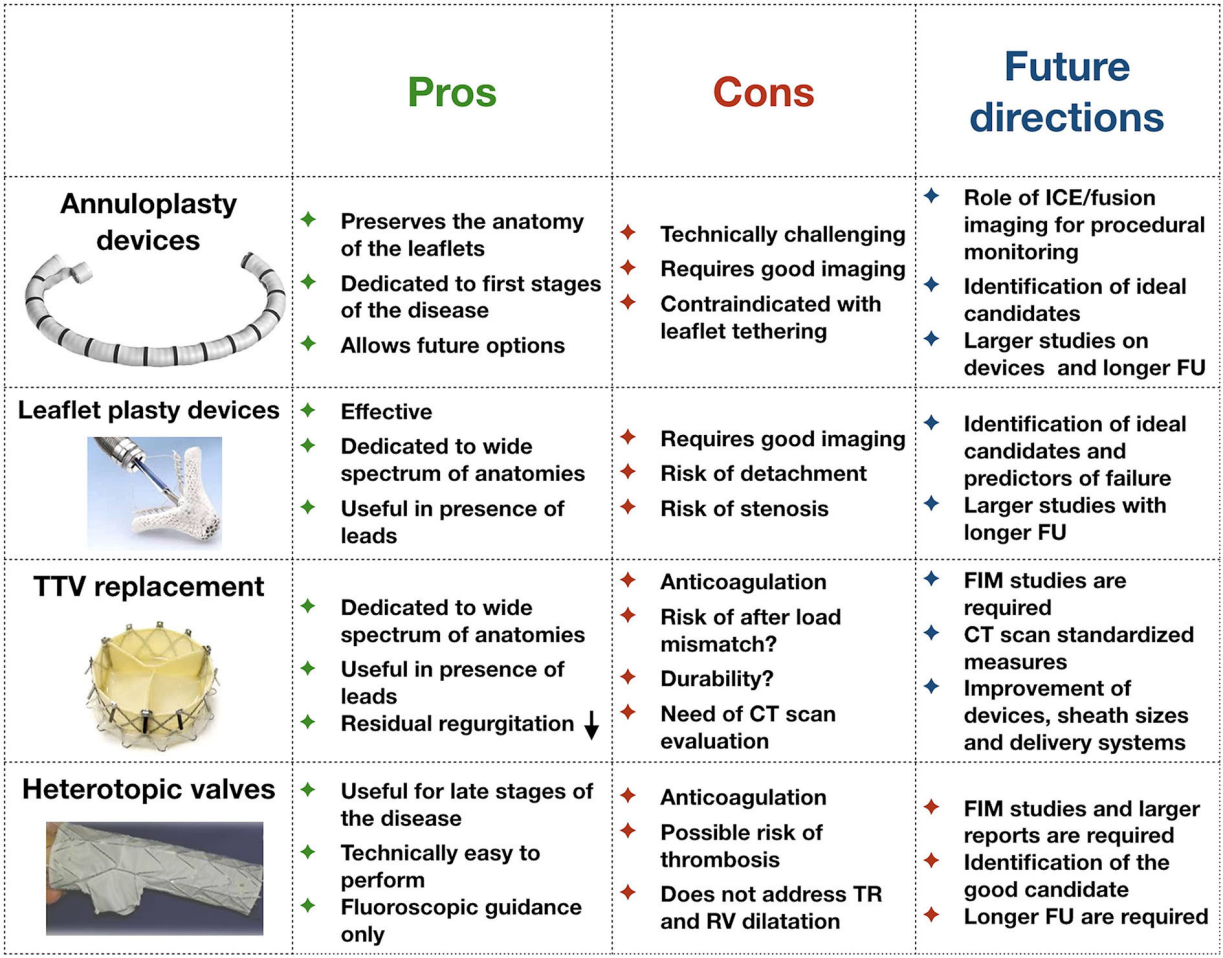
Pros and cons of each kind of technology currently under development for the treatment of tricuspid regurgitation. FU, follow-up; FIM, first-in-man studies; TR, tricuspid regurgitation; CT, computed tomography.

A large number of percutaneous TV devices are currently under development (14). Most currently available devices address the issue of annular dilatation by reducing the antero-posterior and septo-lateral dimensions of the valve. In contrast, the most frequently performed procedure is edge-to-edge repair with MitraClip in tricuspid position (leaflet plasty), probably because of a combination of higher operator experience with the MitraClip device and technical challenge with annuloplasty devices. Other percutaneous solutions target the TR indirectly, by reducing the backflow into the vena cava using valved systems positioned across the right atrium or in the proximal portion of the inferior and superior vein cava. On the other hand, there is great interest in developing fully percutaneous TVs: their appeal is related to the theoretical capacity to abolish the regurgitation in case of optimal positioning. The advantages, disadvantages as well as future perspectives for all these devices are summarized in Figure 2. In particular:

- **Annuloplasty devices:** There is a wide spectrum of annuloplasty devices under pre-clinical and clinical development, that could be categorized as ring-based direct annuloplasty systems (Cardioband [Edwards Lifescience, Irvine, California], Millipede IRIS [Boston Scientific, Marlborough, Massachusetts], DaVingi [Cardiac Implants, Tarrytown, New York]), ring-based indirect annuloplasty systems (transatrial intrapericardial tricuspid annuloplasty [Health and Cook Medical, Bloomington, Indiana]), or direct suture systems (Trialign [Mitralign, Tewksbury, Massachusetts], Tricinch [4Tech Cardio, Galway, Ireland], minimally invasive annuloplasty technology [Micro Interventional Devices, Newtown, Pennsylvania], pledget-assisted suture tricuspid annuloplasty). The Cardioband device was evaluated in the TrIcuspid Regurgitation RePAIr With CaRdioband Transcatheter System (TRIREPAIR) trial and, after the positive results obtained in terms of both procedural success and 6 months follow-up, it received the conformité européenne (CE) mark in the 2019 (15). Although the device was successfully implanted in all patients, 28% of the patients had at least severe or torrential TR (mean vena contracta of 9 mm) at follow-up, despite an overall reduction in the septo-lateral dimensions of 3.8 mm. Future studies are required to better understand which patients are ideal candidates for percutaneous annuloplasty. Patients with leaflet tethering, organic degeneration of the leaflets and TR secondary to partial interference of pacemaker leads are unlikely to derive significant benefit from an annuloplasty strategy (16–18). Echocardiographic predictors are required to discern those who will benefit the most from percutaneous annuloplasty.
  Of note, contemporary devices can result in either complete or incomplete annuloplasty. In the surgical experience, complete annuloplasty is associated with less recurrence of significant TR at follow-up (19). It is possible that incomplete annuloplasty achieved via a percutaneous device could have less efficacy, especially amongst patients with advanced tricuspid annular dilatation. In some cases, this aspect could be related to the commercially available device sizes (e.g., Cardioband implant sizes) and could be potentially overcome in the future. Future studies are needed to address this point.
- **Leaflet devices**. These are the most commonly used devices in clinical practice to percutaneously treat TR, with the TriClip being the device of choice in more recent years (20). It acts by replicating both the surgical clover technique or the Kay bicuspidization of the valve (21). Due to its widespread use, numerous retrospective registries have identified the best anatomical scenario where TriClip can assure good echocardiographic results. The Transcatheter Clip Repair System in Patients With Moderate or Greater TR (TRILUMINATE) trial demonstrated the feasibility of the TriClip in a selected cohort of patients with stable results at 6 months follow-up (15). The PASCAL (Edwards Lifescience, Irvine, CA) clip is an alternative device, which has been used in the tricuspid position with good feasibility, safety and procedural outcomes (22). Other devices still remain under clinical development (14). Similar to annuloplasty devices, future studies are required to identify which patients and specifically anatomies are most suited to treatment with leaflet plasty devices. In particular, leaflet devices could be useful in patients with combined annular dilatation and leaflet remodeling (leaflet tethering) because of their capacity to reshape the TV and indirectly reduce annular dimensions (14, 23). However, the following points need to be addressed in the future:
  ◾ ***Residual regurgitation and how to achieve good procedural results*. **Data from the TRILUMINATE trial indicate that 42% of treated patients have severe TR at 6 months follow-up (24). There is a significant gap in knowledge pertaining to potential predictors of residual TR after use of leaflet plasty devices. Retrospective data indicate that patients with more advanced valve remodeling (large coaptation gap, large tenting area, higher effective regurgitant orifice area) and with regurgitant jets originating from the septal-posterior commissure are less likely to benefit from TriClip intervention in terms of echocardiographic result (25); however, future studies focusing on predictors of procedural failure are needed to optimize procedural results. Moreover, specific delivery catheters are required to facilitate TV intervention.
    The new Triclip has a brand new delivery system and guiding catheter specifically designed for tricuspid intervention. This innovations help the operator to have an easier alignment to the TV and a wider range of maneuvers, thus reducing the procedural time and expanding the possibility of intervention.
  ◾ ***Post-procedural stenosis*. **Leaflet plasty devices have the potential negative effect of creating an iatrogenic reduction in the TV area. Although the risk of significant stenosis is theoretical because of the large area, results from retrospective registries show that a trans-tricuspid gradient >3 mmHg was found in the 17.2% of the patients, albeit without any negative impact on 1 year survival. In the TRILUMINATE trial, 7% of patients had a mean gradient >5 mmHg but without any clinical consequence. However, the issue of iatrogenic TV stenosis needs further investigation with larger future studies.
  ◾ ***Device detachment*. **Leaflet plasty devices improve leaflet coaptation by applying a tension force upon the valve leaflets. This “coaptation force” is distributed along the leaflets and the annulus resulting in a reduction of the valve area and of the annular dimensions. In patients with severe remodeling of the TV, the use of leaflet plasty devices can result in excessive traction along the insertion point of the device with the consequent risk of device detachment. Certain technologies have indeed failed because of dramatic rates of device detachment and second generation devices are being developed to overcome this issue (26). Retrospective registries report a detachment rate of 12.9 and 7.1% with the Mitraclip XTR and PASCAL devices, respectively, whilst in the TRILUMINATE trial (Mitraclip NTR) the rate of single leaflet device attachment was 7% (22, 24, 27). Further evaluation of the causes, mechanisms and subsequent treatment of this condition are required.
- **Transcatheter tricuspid valve replacement (TTVR)**. Pioneering reports have demonstrated that TTVR can potentially abolish TR with an acceptable rate of complications (28, 29). To date, at least 100 cases of TTVR have been performed worldwide and in most of the cases, a direct transatrial access has been chosen (30, 31). Preliminary experience suggests a satisfactory safety profile and good echocardiographic outcomes. Even if this technology seems to be promising, a number of technical issues need to be addressed:
  ◾ ***Need for feasibility and first in man studies*. **Initial studies regarding early experiences with TTV replacement are limited and without any external event adjudication or centralized corelab analysis for echocardiographic outcomes. Need for rigorous evaluation and reporting of outcomes in early feasibility trials is required.
  ◾ ***Prolonged follow-ups*. **Most of the studies on TTVR report short-term, usually 30 days follow-up. Studies evaluating the mid and longer-term performance of these valves are warranted. Moreover, issues pertaining to valve durability, valve thrombosis and appropriate antithrombotic regimen post-implantation remain unresolved.
  ◾ ***Valve sizing and access route*. ***First* generation devices for TTVR are often characterized by the lack of a steerable delivery system, which makes the approach to the TV challenging. Often a transatrial approach or a surgical cutdown even for transjugular and transfemoral access is required. Aspects of valve sizing and prosthesis selection are yet to be determined.
- **Heterotopic valves**. Heterotopic valves were conceived to create a “neovalve” which blocks the backflow and blood stasis related to TR. However, heterotopic valves do not treat TR and do not prevent negative right ventricular remodeling. Few studies are available about the follow-up of patients treated with this technology (32). Even if the procedure is less challenging compared to the aforementioned technologies dedicated to the TV, future studies are required to establish the real benefit of this technology not directly treating the TV disease. Specifically, a persistent symptomatic benefit should be demonstrated, whereas the risk of thrombosis and the optimal antithrombotic regimen for these devices have to be established.

## Cardiac Imaging is the Key for the Future of Transcatheter Tricuspid Interventions

A comprehensive knowledge of TV anatomy and imaging techniques are of paramount importance for the interventional cardiologist focusing on the percutaneous treatment of TR. Multimodality imaging is essential to refine TR diagnosis and grading, optimize transcatheter device selection, guide device implantation during the procedure, and assess residual TR at follow-up.

Transthoracic echocardiography and transesophageal echocardiography (TEE) represents the primary imaging modality used for TR detection, identification of mechanism and grading, evaluation of feasibility for different percutaneous interventions, intraprocedural guidance, and follow-up (33, 34). Besides its well-established role in TR identification and grading (35), echocardiography also enables a comprehensive assessment of right ventricular dimensions and function, estimation of pulmonary artery pressures, and evaluation of left heart structures to be undertaken. These additional evaluations are fundamental in the pre-procedural screening of candidates for TTVIs (34).

Intraprocedural echocardiographic monitoring is crucial for the success of percutaneous TV interventions, and requires a multimodality approach combining 2D and 3D dedicated transesophageal windows, fluoroscopy, angiography, and fusion imaging tools (36, 37). Dedicated fluoroscopic views with optimal angles for intraprocedural guidance can be derived from pre-procedural multislice computed tomography (MSCT) scans, and integrated with specific modified TEE views to facilitate transcatheter TV interventions (38, 39). Furthermore, intracardiac echocardiography (ICE) represents a particularly interesting tool in the percutaneous TV field, given its ability to visualize right-sided structures (TV apparatus and right ventricle) alongside its established general advantages (no strict need of general anesthesia, potentially performed by a single operator, providing high-resolution imaging) (40–42). Since its first clinical application in 2013 (43), 3D ICE is developing into a valid alternative to 3D TEE (40). Currently, 3D ICE technology is still at an early stage, and the only available ICE catheter with 3D capabilities (AcuNav-V [Siemens-Acuson, Mountain View, CA]) has several technical limitations, which limit its incremental value over traditional 2D ICE and does not replicate the imaging accuracy of 3D TEE. Future iterations of this catheter and development of new dedicated catheters are expected to expand the role of 3D ICE in the guidance of percutaneous structural interventions, potentially limiting the need for TEE (and, subsequently, general anesthesia with endotracheal intubation) without impacting procedural results (40).

Besides intraprocedural guidance during transcatheter TV interventions, multimodality imaging also plays a key role in pre-procedural planning. A combination of both echocardiographic evaluation of TR mechanism and grading with MSCT scan evaluation of TV annulus, distance between TV annulus to RV apex, and localization of the right coronary artery and coronary sinus, is particularly useful before intervention (44, 45). The role of MSCT imaging in patient screening and pre-procedural planning is important for both transcatheter TV repair and replacement procedures.

Similar to detailed pre-procedural MSCT screening protocols prior to transcatheter aortic valve replacement, standardized acquisition protocols and measurements are crucial when performing and analyzing MSCT before TV interventions (46). Indeed, the assessment of safety issues is fundamental before several percutaneous TV repair interventions (evaluation of TV annulus size, distance between TV annulus and right coronary artery, distance between TV annulus and RV apex, RV size, right atrium size, localization of coronary sinus) as well as the development of a standardized measurement protocol is necessary before TTVR.

## Conclusions

TTVI is a fast-growing field in interventional cardiology set to expand dramatically over the next few years. Procedural success is likely to improve in the near future as these technologies expand toward lower-risk populations in whom the underlying valve and ventricular anatomy and physiology is less severe.

At present, many unanswered questions remain regarding patient and device selection, anatomical eligibility, optimal procedural timing, antithrombotic therapy, and imaging: these issues will be the subject of further investigation in the coming years. To facilitate this advancement in knowledge, standardized definitions for reporting procedural and clinical outcomes are required in order to better assess the efficacy of transcatheter therapies compared to medical therapy and surgical interventions.

## Author Contributions

All authors significantly contributed to this work and approved the final version of the manuscript.

## Conflict of Interest

AM had served on the advisory board of Boston Scientific. FG was proctor for Neovasc. AL had served on the advisory boards of Medtronic, Abbott Vascular and Edwards Lifesciences. The remaining authors declare that the research was conducted in the absence of any commercial or financial relationships that could be construed as a potential conflict of interest.

## Abbreviations

ICE: intracardiac echocardiography
TV: tricuspid valve
TR: tricuspid regurgitation
TTVI: transcatheter tricuspid valve intervention
TTVR: transcatheter tricuspid valve replacement
TTE: transesophageal echocardiography
MSCT: multislice computed tomography.
